# Population prevalence and distribution of ankle pain and symptomatic radiographic ankle osteoarthritis in community dwelling older adults: A systematic review and cross-sectional study

**DOI:** 10.1371/journal.pone.0193662

**Published:** 2018-04-30

**Authors:** Charlotte Murray, Michelle Marshall, Trishna Rathod, Catherine J. Bowen, Hylton B. Menz, Edward Roddy

**Affiliations:** 1 Research Institute for Primary Care & Health Sciences, Keele University, Keele, Staffordshire, United Kingdom; 2 Faculty of Health Sciences, University of Southampton, Southampton, United Kingdom; 3 School of Allied Health, La Trobe University, Bundoora, Victoria, Australia; University of Umeå, SWEDEN

## Abstract

**Objectives:**

To identify by systematic review published prevalence estimates of radiographic ankle osteoarthritis (OA) and to subsequently estimate the prevalence of ankle pain and symptomatic, radiographic ankle OA within community-dwelling older adults from North Staffordshire, UK.

**Methods:**

Electronic databases were searched using terms for ankle, osteoarthritis and radiography. Data regarding population, radiographic methods, definitions and prevalence estimates of ankle OA were extracted from papers meeting predetermined selection criteria. Adults aged ≥50 years and registered with four general practices in North Staffordshire were mailed a health questionnaire. Ankle pain in the previous month was determined using a foot and ankle pain manikin. Respondents reporting pain in or around the foot in the last 12 months were invited to attend a research clinic where weight-bearing, antero-posterior and lateral ankle radiographs were obtained and scored for OA using a standardised atlas. Prevalence estimates for ankle pain and symptomatic, radiographic ankle OA were calculated using multiple imputation and weighted logistic regression, and stratified by age, gender and socioeconomic status.

**Results:**

Eighteen studies were included in the systematic review. The methods of radiographic classification of ankle OA were poorly reported and showed heterogeneity. No true general population prevalence estimates of radiographic ankle OA were found, estimates in select sporting and medical community-dwelling populations ranged from 0.0–97.1%. 5109 participants responded to the health survey questionnaire (adjusted response 56%). Radiographs were obtained in 557 participants. The prevalence of ankle pain was 11.7% (10.8,12.6) and symptomatic, radiographic ankle OA grade≥2 was 3.4% (2.3, 4.5) (grade≥1: 8.8% (7.9,9.8); grade = 3: 1.9% (1.0,2.7). Prevalence was higher in females, younger adults (50–64 years) and those with routine/manual occupations.

**Conclusion:**

No general population prevalence estimates of radiographic ankle OA were identified in the published literature. Our prevalence study found that ankle pain was common in community-dwelling older adults, whereas moderate to severe symptomatic, radiographic ankle OA occurred less frequently. Further investigations of the prevalence of ankle OA using more sensitive imaging modalities are warranted.

## Introduction

Osteoarthritis (OA) is a recognised global burden of disease, affecting more than 100 million people worldwide [1]. The prevalence of symptomatic, radiographic OA in adults aged 60 years and over is reported to be 10% for men and 18% for women, and as populations age, it is predicted that OA will become one of the leading causes of disability worldwide by the year 2020 [2]. OA at joint sites such as the knee, hip and hand have received considerable attention, in comparison, the foot and ankle joints have been relatively neglected. The prevalence of ankle OA within the general population is not clear, but clinical observations and experience suggest that it is significantly lower than OA of the knee or hip [3]. Estimates of the prevalence of ankle pain range from 9% to 15% in general adult populations [4,5].

Previous reviews have examined the methods of radiographic assessment at the knee, hip, hand and foot [6, 7, 8, 9]. However, the methods used to examine radiographic ankle OA and define its presence have not been reviewed previously, and estimates of ankle OA have ranged widely between 0 and 97% [10,11]. The variations between studies regarding the methods and definitions used for radiographic ankle OA, as well as the characteristics of the study populations examined, may explain the differences in reported prevalence estimates between studies.

While the population prevalence of ankle pain within the general population has been estimated previously [4, 5, 12, 13], there seems to be a paucity of existing studies examining radiographic ankle OA in community-dwelling populations. Population prevalence estimates for both ankle pain and symptomatic, radiographic ankle OA would demonstrate the burden of these two conditions within a community setting more clearly and inform healthcare provision and clinical needs. It would also provide a basis for understanding better the aetiology of ankle OA and the association between ankle pain and the occurrence of OA.

The objective of this study was (i) to undertake a systematic review to identify existing prevalence estimates for radiographic ankle OA in community-dwelling populations and (ii) to estimate the prevalence of ankle pain and symptomatic radiographic ankle OA in a population of community-dwelling older adults aged 50 years and over.

## Materials and methods: Systematic review

### Search strategy

A search strategy was developed in order to identify all community-dwelling epidemiological studies which examined radiographic ankle OA in adults. Electronic searches were conducted in Medline, Embase and CINAHL and all studies registered on these databases from inception until the search date (31^st^ December 2016) were eligible for inclusion in this review. The search strategy used the subject index terms ‘osteoarthritis’, ‘radiology’ ‘radiography’, ‘x-ray’, ‘x-ray film’ and ‘ankle’ as well as the following free text terms: osteoarthr* or degenerative arthr* or OA AND radiograph* or radiolog* or x ray or imaging or rontgen* or roentgen* AND ankle or ankle joint or talus or talar or talocrural or talotibial or talofibul* or tibiotal* or tibiofibul* or hindfoot or hind foot). This review was conducted and reported in line with the Preferred Reporting Items for Systematic Reviews and Meta-Analyses (PRISMA) statement (S1 File).

### Search criteria

A single reviewer (CM) screened the titles and abstracts using the following selection criteria. The inclusion criteria were: (1) human subjects, (2) the study population contained adults aged 18 years and over, (3) plain film radiography was used to assess the presence of ankle OA, (4) the study population was from a community-dwelling or population. Exclusion criteria were: (1) non-human studies, (2) studies that involved solely children (<18 years), (3) studies that used only macro radiography, computerised tomography, magnetic resonance imaging, ultrasound or scintigraphy, (4) studies that recruited patients from secondary or tertiary care settings, (5) clinical trials or narrative reviews, (6) incomplete or un-published articles.

### Data extraction and synthesis

Titles and abstracts not excluded or those whose relevance was ambiguous were retained for full-text review. A single reviewer (CM) assessed the full texts and conferred with two other researchers (ER, MM) when uncertainty remained. Two non-English articles were identified in the search and translations were obtained. Additionally, reference lists of papers included in the review were also screened for further relevant articles.

Data extracted from all papers that satisfied the selection criteria included the following information: study population and demographics, anatomical definition of the ankle joint, radiographic views, method of radiographic assessment of ankle OA including reliability, definition of radiographic ankle OA and prevalence estimates of radiographic ankle OA. Quality assessment was performed using the 8-item Newcastle-Ottawa Quality Appraisal Scale, by a single reviewer (CM) who conferred with two other researchers (ER, MM) when uncertainty remained [14]. The scale was modified to 5-items for cross-sectional studies questions by dropping questions related to longitudinal study design.

## Results: Systematic review

### Search

**Fig 1:**
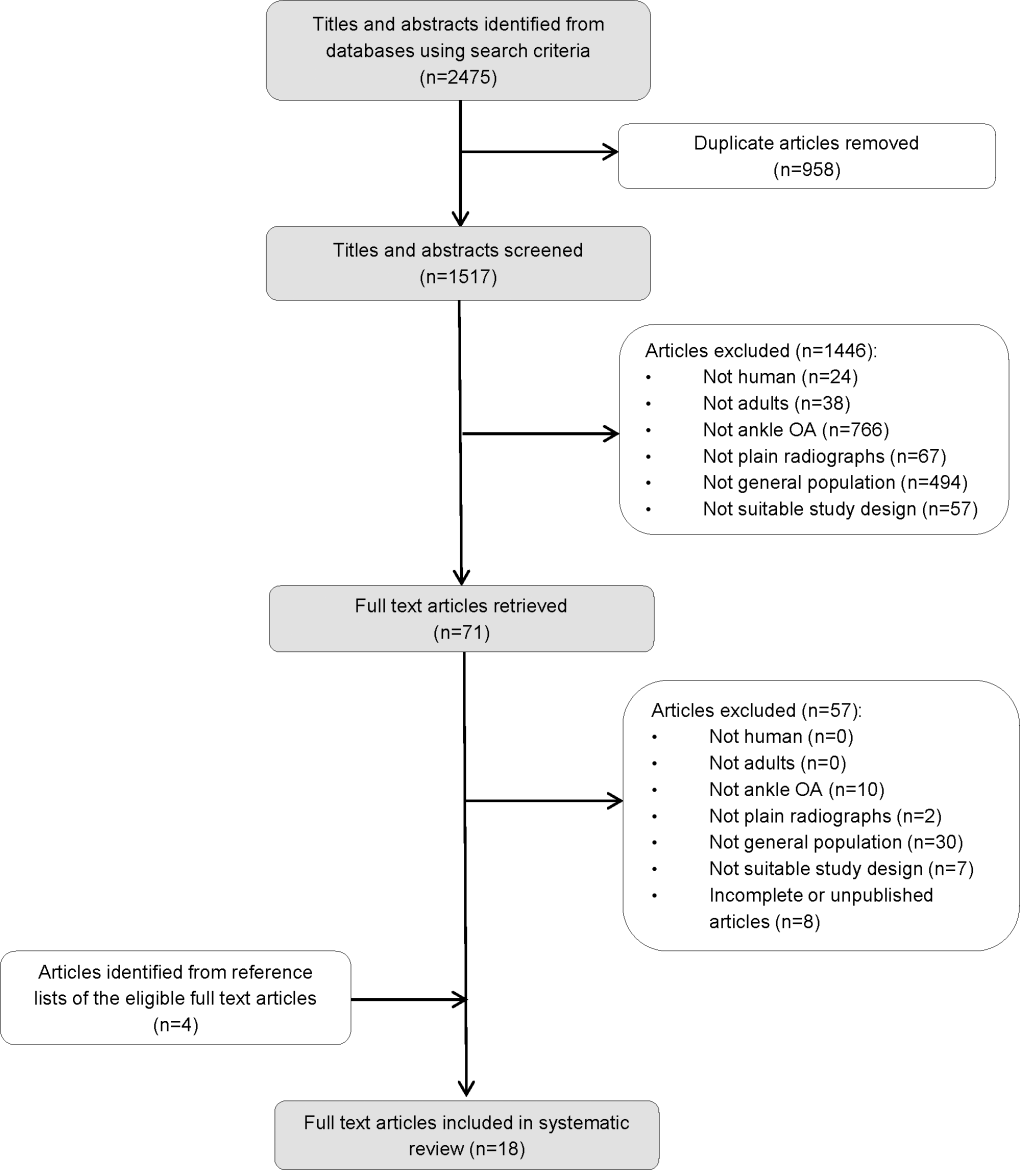
Flow diagram of publications included and excluded at each stage of the review.

The search identified 1517 unique papers of which 1446 were excluded through review of titles and abstracts (Fig 1). After full-text review of the remaining 71 publications, a further 57 were excluded, leaving 14 papers to be included. Screening the reference lists of these papers identified a further four studies suitable for inclusion. Therefore, a total of 18 papers were included [10,11,15–30].

### Quality appraisal

Quality appraisal using the Modified Newcastle Ottawa Scale Score demonstrated that no studies were of exceptionally high quality (Table 1). All studies assessed ankle OA radiographically however, the majority of studies were limited by being undertaken in populations selected specific from sporting groups or with a particular medical condition (n = 15) [10,11,15–26,30].

**Table 1:** Description of the populations and the radiographic assessment undertaken in publications included in the review.

Study	Population	Newcastle Ottawa Scale score†	Sample size	Age range in years (mean)	% Female	Joints examined	Radiographic views	Radiographic assessment	Reliability	Ankle OA definition	Prevalence estimate of ankle OA
Adams, 1979 [24]	Football club; Leeds, UK	1/5	62	15–55 (23)	NR	Tibia and talar surfaces	NWB, AP & LAT	IRF (P/A): OST, JSN, LB, NBF	NR	Presence of JSN & NBF	1.6%
Andersson et al., 1989 [23]	Retired ballet dancers; Sweden; Norway; Copenhagen, Denmark	1/5	44	44–80 (57)	66%	Ankle	NWB, AP & LAT	IRF (P/A): JSN	NR	Presence of obvious JSN	2.3%
Armenis et al, 2011 [15]	Former elite football players aged >40 years & gender matched non-sporting controls; Athens, Greece	2/5	182	42–55 (50)	0%	Ankle	WB, AP	IRF (P/A): OST, JSN, SCL, CYT	NR	NR	Former football players: 8.8%, Control population: 3.7%
Brodelius, 1961 [11]	Football players, ballet dancers & patients examined with a foot injury; Malmo, Sweden	1/5	245	18–46 (NR)	35%	Talar joints	NR, NR	NR	NR	NR	Football players: 97.1%, Ballet dancers: 87.5%, Control patients: <24 years 3.0% & >45 years 50.0%
Carroll et al., 2011 [27]	Individuals from general population ≥40 years with hereditary haemochromatosis; Western Australia	1/5	103	41–83 (NA)	58%	Ankle	NR, AP & LAT	Kellgren & Lawrence score	NR	Grade ≥2 in both ankles	1.9%
Gross & Marti, 1999 [18]	Former league volleyball players & normal healthy gender matched controls; Magglingen, Sweden	2/5	41	NR (35)	0%	Ankle	WB, AP	IRF: OST (0–3), JSN (0–3), SCL talar & tibial (0–3)	NR	Sum score for IRF >2	Volleyball players: 86.4%, Untrained males: 22.2%
Iosifidis et al., 2015 [30]	Former elite male athletes and controls; Greece	2/5	335	NR (50)	0%	Ankle	WB, NR	Kellgren & Lawrence score	NR	Grade ≥2	Former athletes: 7.0%, Controls: 3.7%
Jenyo et al., 2014 [29]	Primary care patients with joint symptoms; Osun State, Nigeria	2/5	90	20–60 (NR)	60%	Ankle	NR, NR	NR	NR	NR	NR
Knobloch et al., 1990 [21]	Former national team long distance runners, orienteers, bobsledders & healthy individuals matched by gender recruited from a previous RCT; Switzerland	6/9	50	33–46 (39)	0%	Ankle	WB, AP	Bargon arthrosis score (modified; 0–4)	NR	Grade ≥3	Runners & orienteers: 59.3%, Bobsledders: 44.4%, Healthy individuals: 26.3%
Konradsen et al., 1990 [22]	National orienteers & non-running patients referred for abdominal radiology exams matched for age, weight, height and physical work load; Denmark	3/5	54	50–68 (58)	0%	Ankle	WB, AP & LAT	IRF (P/A): OST, Cartilage thickness (mm)	NR	Cartilage thickness ≤3mm	NR
Muehleman et al., 1997 [28]	Cadavers; Chicago, USA	2/5	50	36–94 (76)	52%	Talocrural	NR, NR	NR	NR	NR	NR
Murray-Leslie et al., 1977 [25]	Ex-military and sport parachutists; Leeds, UK	1/5	221	23–70 (38)	5%	Talotibial	NR, AP	IRF: OST (0–4), JSN (0–4)	NR	Grade >2 for OST or JSN	17.5%
Panush et al., 1986 [10]	Runners aged >50 years weekly distance ≥32km (20 miles) for ≥5 years & non-runners with normal body weight; Florida, USA	2/5	35	50–74 (59)	0%	Ankle	NR, AP & LAT	IRF (P/A): OST, Cartilage thickness (mm)	NR	Cartilage thickness ≤3mm	Runners: 0.0%, Non-runners: 0.0%
Schmitt et al., 2003 [17]	Former male high jump athletes & age & BMI matched male controls; Heidelberg, Germany	3/5	40	32–56 (42)	0%	Talotibio fibular	NR, NR	Bargon arthrosis score (0–3), Scranton & McDermott score (I-IV)	Inter-rater: K = 0.57–1.00,Intra-rater: NR	NR	NR
Schmitt et al., 2004 [16]	Former long and triple jump athletes; Germany	3/9	29	36–59 (44)	0%	Ankle	NR, AP & LAT	Bargon arthrosis score (0–3), Scranton & McDermott score (I-IV)	Inter-rater: K = 0.57,Intra-rater: K = 1.00	Bargon score ≥1, Scranton & McDermott score ≥2	Bargon score: Push-off leg = 75.0%,Swing leg = 67.9%; Scranton & McDermott score: Push-off leg = 39.3%, Swing leg = 39.3%
Teitz & Kilcoyne, 1998 [19]	Former professional dancers for >10 years & age matched non-dancers with lower limb injuries or pain; Washington, USA	2/5	50	27–46 (35)	64%	Ankle	NR, NR	IRF (P/A): OST, JSN, SCL, CYT	NR	NR	Professional dancers: 50.0%, Non-dancers: 0.0%
Van Dijk et al., 1995 [20]	Former professional dancers aged 50–70 years & gender, age, height & weight matched outpatients with no lower limb complaints; Amsterdam, Netherlands	3/5	38	50–66 (59)	100%	Ankle	NR, NR	IRF (P/A): OST, SCL, CYT, BD, JSW (mm), Hermodsson scale (modified, 0–3)	NR	NR	NR
Vincelette et al., 1972 [26]	Football players & unspecified gender matched control population; Montreal, Canada	3/5	109	19–30 (23)	0%	Ankle	NR, NR	IRF (P/A): OST, SCL, Irregular joint line	NR	Mild OA = 1 IRF, Severe OA = 2–3 IRFs	Football players: mild = 30.0%, severe = 63.0%; Controls: mild = 6.0%, severe = 0.0%

† The Newcastle-Ottawa quality assessment scale was marked out of nine for cohort studies (with 1 item contributing 2 points) and modified and marked out of five for cross-sectional studies with higher scores indicating more rigorous methodological design.

OA: Osteoarthritis; NR: Not reported; NA: Not applicable; WB: Weight-bearing; NWB: Non weight-bearing; AP: Anterior-Posterior View; LAT: Lateral View; IRF: Individual Radiographic Features; P/A: Presence/absence; OST: Osteophytes; JSN: Joint Space Narrowing; LB: Loose Bodies; NBF: New Bone Formation; SCL: Sclerosis; CYT: Cysts; JSW: Joint Space Width; BD: Bone Destruction; K: Kappa.

### Study population

Sample sizes were frequently small: nine studies had ≤50 participants [10,16–21,23,28], three studies had 51–100 [22,24,29] and six studies had >100 [11,15,25–27,30]. The study populations were frequently specific sub-groups within a community setting rather than representing true general populations (Table 1). No study reported general population prevalence estimates for radiographic ankle OA. Fifteen studies used select sporting populations [10,11,15–26,30], one used a population with hereditary haemochromatosis [27], one utilised human cadavers [28] and one involved a small urban population in Nigeria who were attending a primary care facility with joint symptoms [29]. All studies included adults although these were frequently younger adults: in two studies age range was not reported but mean age was 35 years [18] and 50 years [30], six studies reported age range with an upper limit of 55 years [11,15,19,21,24,26], ten studies showed an age range which extended past 55 years [10,16,17,20,22,23,25,27–29]. Nine studies examined exclusively male populations [10,15–18,21,22,26,30] and one study examined an exclusively female population [20]. One study did not report the gender of their population [24].

### Joints examined and radiographic views

Most studies broadly described studying the ‘ankle joint’ while five specifically described the exact regions of the ankle that were examined radiographically for OA and variations were present between these studies (Table 1) [11,17,24,25,28]. Eight studies failed to provide details of the radiographic views that were used to assess ankle OA [11,17,19,20,26,28–30]. The remaining ten all included an anterior-posterior view with six also obtaining a lateral view [10,15,16,18,21–25,27]. Seven studies provided information regarding whether views were weight-bearing [15,18,21–24,30]. The radiographic assessment technique was not described in three studies [11,28,29].

Ten studies graded individual radiographic features to assess the presence of OA [10,15,18–20,22–26]: nine of these studies graded osteophytes [10,15,18–20,22,24–26], seven joint space narrowing/width [15,18–20,23–25] and five subchondral sclerosis [15,18–20,26]. The six remaining studies used established grading systems: the Kellgren and Lawrence scoring system (0–4) [27,30,31], the Scranton and McDermott Score (I-IV) [16,17,32], the modified Hermodsson Scale [20], and the unmodified [16,17,33] and modified [21] Bargon Arthrosis Score (Table 1). None of the studies that assessed radiographic ankle OA included symptoms in their definitions of ankle OA. Reliability of the radiographic assessment of ankle OA was assessed and reported in only two studies [16,17].

### Prevalence estimates of radiographic ankle OA

Thirteen publications reported prevalence estimates for radiographic ankle OA ranging from 0.0% populations of runners, non-runners and those with reported lower limb injuries or pain [19,10] to 97.1% in a population of football players [11] however, none of these were representative of a general population (Table 1) [10,11,15,16,18,19,21,23–27,30]. Eight publications presented comparison estimates between select populations and a control group, with higher estimates for the selected population compared to the control group in each study [10,11,15,18,19,21,25,30]. In only one study were the prevalence estimates for radiographic ankle OA stratified by age and then only for the control group [11]. As a result of this, it was not possible to undertake a meta-analysis to calculate a pooled prevalence estimate.

## Materials and methods: Prevalence study

### Study design and data collection

This study used baseline cross-sectional data collected from a prospective population-based cohort study, the Clinical Assessment Study of the Foot (CASF) [34,35]. A total of 9194 adults aged 50 years and over registered with four general practices in North Staffordshire, UK, irrespective of consultation for foot or ankle problems, were mailed a baseline health survey questionnaire which collected the following information: demographic, socioeconomic, general health, anthropometric measurements, Short Form 12 (SF-12) [36], Hospital Anxiety and Depression Scale (HADS) [37], details of foot and ankle pain including duration of foot pain in the last one year, the Manchester Foot Pain and Disability Index (MFPDI) pain and functional limitation subscales [38] and a foot pain manikin asking areas of pain in the past one month to be shaded (The University of Manchester 2000. All rights reserved.) [39]. All participants provided written informed consent and ethical approval was obtained from the Coventry Research Ethics Committee, UK (REC reference number: 10/H1210/5).

Participants who reported experiencing pain in or around the foot within the last 12 months and who consented to further contact were invited to attend a research clinic for further clinical assessment and radiographic imaging [34]. At the research clinic, weight-bearing, antero-posterior radiographs of both ankle joints and lateral views of both feet were obtained according to a standardised protocol [34].

Radiographic grading of ankle OA assessed the articulation between the talus, tibia and fibula. A single blind assessor used a standardised atlas (developed as an extension of one recently developed for the foot, due to the lack of an appropriate scoring system for assessing individual radiographic features in ankle OA at the time) [40], where the presence of osteophytes and joint space narrowing were graded on a scale of 0–3 in both views. The presence of osteophytes at the ankle joint was graded as absent (score = 0), small (score = 1), moderate (score = 2) or severe (score = 3), and presence of joint space narrowing was graded as none (score = 0), definite (score = 1), severe (score = 2), or joint fusion at one point or more (score = 3). Radiographic images for the standardised classification atlas were assessed by three of the authors (radiographer MM, rheumatologist ER and podiatrist HBM) for possible representativeness of each grade (0–3) for osteophytes and joint space narrowing from which a provisional atlas was compiled. This provisional atlas was reviewed by the development team (MM, ER and HBM) and where an image was deemed unsuitable due to the presence of other OA features or imprecise patient positioning, it was replaced with another that was considered to be a better representation. This process was repeated until there was consensus amongst the team for every image representing each of the grades (0–3) for both of the OA features. In order to confirm quality and clinical relevance, the collection of images for inclusion in the ankle atlas were then reviewed by a specialist consultant musculoskeletal radiologist (Dr J Saklatavala) before the Radiographic Classification Atlas of Ankle Osteoarthritis was finalised (S2 File).

Radiographic ankle OA was defined as a grade ≥2 for either osteophyte or joint space narrowing on either view. Intra-rater reliability of the presence of ankle OA was examined by the assessor (MM) rescoring 60 randomly selected radiographs after a period of at least 8 weeks. Inter-rater reliability was examined by a second blind assessor (HBM) scoring the same 60 randomly selected radiographs. Intra-rater reliability for the presence of ankle OA was shown to be excellent (kappa = 0.87, 95% exact agreement) and inter-rater reliability was found to be fair (kappa = 0.30, 65% exact agreement). An independent third blind assessor (CB) was subsequently asked to score the radiographs and inter-rater reliability remained low with both MM (kappa = 0.18, 60% exact agreement) and HBM (kappa = 0.30, 65% exact agreement).

### Case definitions and exclusions

Ankle pain was defined as pain in the past month indicated by shading of the ankle area on the dorsal view of a foot pain manikin [39]. Symptomatic radiographic ankle OA was defined as the presence of ankle pain and radiographic ankle OA (grade≥2 for either osteophytes or joint space narrowing on either view) in the same ankle.

Participants with a history of inflammatory arthritis (rheumatoid arthritis, psoriatic arthritis or non-specific inflammatory arthritis) identified in their medical records (primary care or local hospital) or in the clinical x-ray report by a consultant musculoskeletal radiologist were excluded from the analyses.

### Statistical analysis

To estimate the prevalence of ankle pain and symptomatic radiographic ankle OA, multiple imputation combined with weighted logistic regression modelling was used. Analysis of the CASF study has previously shown that while responders to the baseline health survey questionnaire were broadly representative of the baseline eligible population, there was selective non-participation in the research clinics [35]. Older women (aged 75 years and over) were under-represented whilst those who attended higher education, were of a professional or managerial occupational class, had a higher number of days with foot pain and greater functional impairment on the MFPDI were more likely to attend the research clinics. Multiple imputation was used to impute missing data that arose from non-completion of individual items or questionnaire non-response. Missing data were associated with a number of variables and therefore data were assumed to be missing at random. The imputation model included the following variables, which included those associated with missing data: age, gender, GP practice, marital status, higher education, employment status, socioeconomic class, SF-12 score, HADS score, reported foot pain in last month, duration of foot pain in last 12 months and Rasch-transformed MFPDI pain and function scores. The MFPDI has previously been shown to fit the Rasch model, allowing interval-level scores to be generated for the pain and function subscales from the instrument that has ordinal responses, so that severity of pain and disability can be more precisely described [41]. The number of imputations was set at 15 and the imputed datasets were combined using Rubin’s rules [42]. The mim:proportion command was used to determine the prevalence estimates and 95% confidence intervals for all responders to the baseline health survey questionnaire.

Prevalence estimates were then weighted to account for any differences in age, gender and GP practice between responders and non-responders to the questionnaire. Logistic regression modelled for each person the probability of completing the questionnaire based on their age, gender and general practice. The inverse of the probability was used to obtain weighted prevalence estimates (and 95% confidence intervals) in the total baseline eligible mailed population from North Staffordshire. Population prevalence estimates for ankle pain and symptomatic radiographic foot OA were stratified for age, gender and socioeconomic status. Sensitivity analysis using different severity thresholds of radiographic OA that included grade≥1 and grade = 3 were also examined using the same processes of multiple imputation and weighted logistic regression. All analyses were undertaken using STATA version 12.0 (Stata Corporation, Texas, USA).

## Results: Prevalence study

### Study population

**Fig 2:**
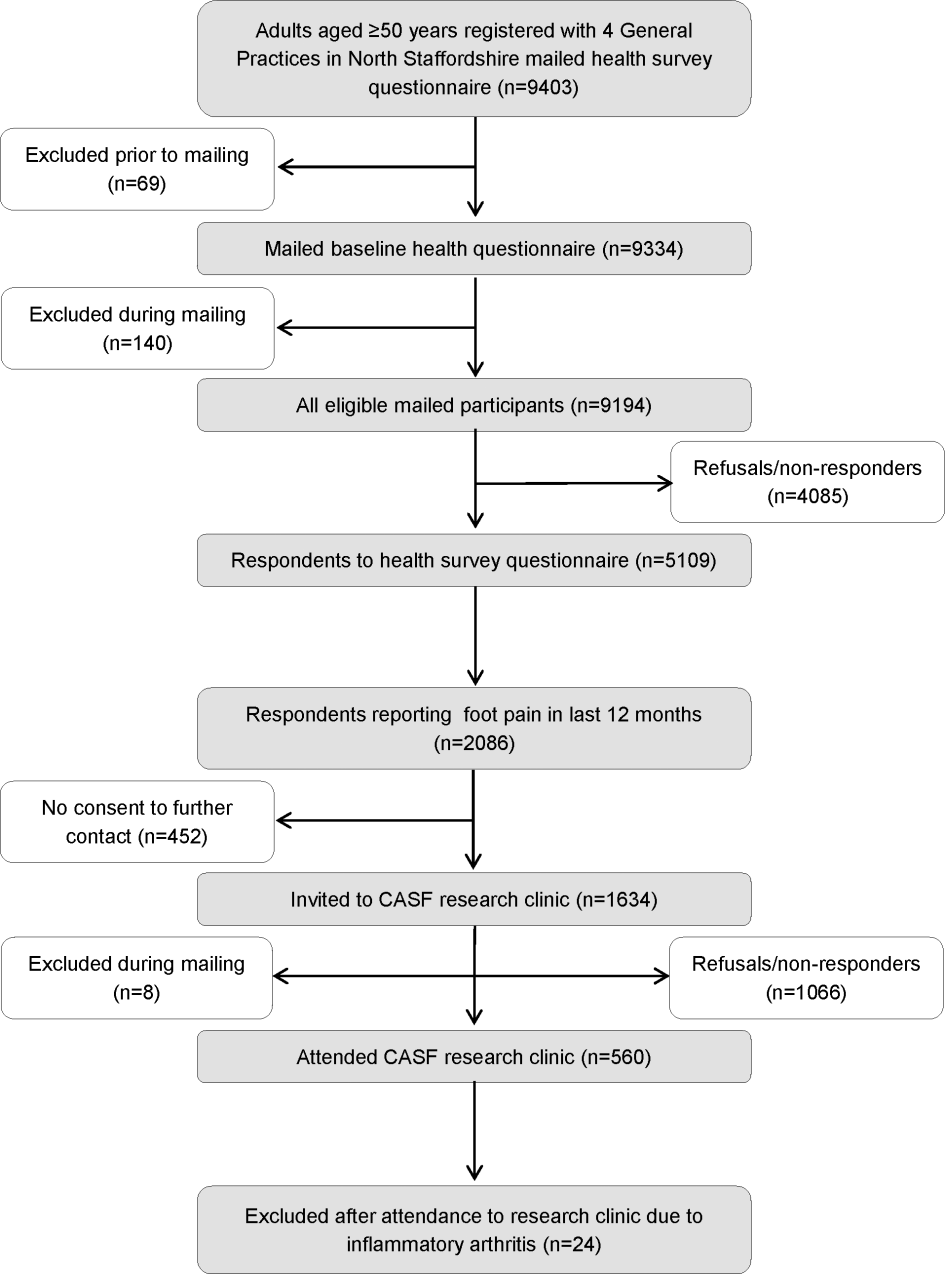
Flow diagram showing CASF study recruitment.

The CASF study identified a total of 9403 adults aged 50 years and over within four GP practices in North Staffordshire however, following exclusions prior to and during mailing, a total of 9194 mailed participants were determined to be eligible. In total, there were 5109 responders to the baseline health survey questionnaires (adjusted response 56%), with 2086 of these (41%) reporting pain in or around the foot in the last 12 months (Fig 2). 1634 of these provided consent to further contact and were invited to attend a research clinic. A total of 560 participants attended the research clinics (adjusted response rate 34%). Participants with inflammatory arthritis were excluded from this analysis (n = 24). Of the 536 eligible individuals without inflammatory arthritis who attended the research clinic, eight had incomplete ankle pain data and four had incomplete radiographic data. Full details of the reasons for exclusion and refusals to take part have been detailed elsewhere [35]. Demographic characteristics of responders and selective non-participation have also been discussed previously [35].

### Prevalence of symptomatic radiographic ankle OA

In the clinic attenders, 31.9% (n = 167) had ankle pain and 7.4% (n = 39) had symptomatic radiographic ankle OA. Using multiple imputation in these individuals allowed the frequency of ankle pain and symptomatic radiographic ankle OA to be determined for the responder population. This showed the prevalences of ankle pain to be 11.6% (95%CI 10.8, 12.5) and symptomatic radiographic ankle OA to be 3.3% (2.4, 4.3).

To extrapolate these results to the total eligible baseline population of community-dwelling adults aged 50 years and over in North Staffordshire, weighted logistic regression was performed using the variables age, gender and general practice which were known for all individuals. The prevalence of ankle pain was 11.7% (10.8, 12.6) and symptomatic radiographic ankle OA, 3.4% (2.3, 4.5).

### Stratification of prevalence estimates

Both ankle pain and symptomatic radiographic ankle OA were more prevalent in females and in younger individuals aged 50–64 years (Table 2). However, there was considerable overlap of the confidence intervals for the prevalence estimates for the different age groups and genders. Individuals identified as having routine or manual occupations demonstrated higher prevalence estimates of both ankle pain and symptomatic radiographic ankle OA, which remained the case after further sub-stratification by age and gender (Table 3).

**Table 2:** Population prevalence estimates of ankle pain and symptomatic radiographic ankle OA, overall and stratified by age and gender.

	Proportion estimate for ankle pain: % (95% CI)	Proportion estimate for SR ankle OA: % (95% CI)
**Overall**	11.7 (10.8, 12.6)	3.4 (2.3, 4.5)
**Gender:**		
Males	9.2 (8.0, 10.4)	2.9 (1.9, 3.9)
Females	14.1 (12.8, 15.5)	3.9 (2.3, 5.4)
**Age (years):**		
50–64	12.2 (10.9, 13.5)	3.6 (2.4, 4.8)
65–74	11.2 (9.6, 12.8)	3.2 (1.8, 4.7)
≥75	11.1 (9.1, 13.0)	3.1 (1.6, 4.6)
**Males by Age (years):**		
50–64	10.3 (8.6, 12.0)	3.4 (2.0, 4.7)
65–74	7.7 (5.8, 9.7)	2.4 (0.9, 3.9)
≥75	7.8 (5.2, 10.5)	2.3 (0.4, 4.3)
**Females by Age (years):**		
50–64	14.1 (12.2, 16.1)	3.8 (2.0, 5.6)
65–74	14.7 (12.1, 17.2)	4.1 (1.8, 6.5)
≥75	13.5 (10.7, 16.9)	3.6 (1.5, 5.8)

CI: Confidence interval; SR: Symptomatic radiographic; OA: Osteoarthritis.

**Table 3:** Population prevalence estimates of ankle pain and symptomatic radiographic ankle OA, stratified by socioeconomic status and age and gender.

	Proportion estimate for ankle pain: % (95% CI)	Proportion estimate for SR ankle OA: % (95% CI)
**Socioeconomic status**		
Managerial & professional	7.6 (5.9, 9.3)	2.4 (1.0, 3.7)
Intermediate	10.9 (8.8, 13.0)	3.0 (1.4, 4.5)
Routine & manual	13.0 (11.7, 14.3)	4.1 (2.6, 5.6)
Other*	13.9 (10.9, 16.9)	2.5 (-0.5, 5.6)
**Males by socioeconomic status**		
Managerial & professional	5.2 (3.4, 7.1)	1.5(0.1, 2.9)
Intermediate	8.9 (6.1, 11.7)	3.1 (1.0, 5.3)
Routine/ manual	10.6 (8.8, 12.3)	3.6 (2.1, 5.2)
Other*	12.1 (7.1, 17.1)	1.9 (-1.3, 5.1)
**Females by socioeconomic status**		
Managerial & professional	10.9 (7.9, 13.9)	3.5 (1.3, 5.7)
Intermediate	12.7 (9.7, 15.8)	2.8 (0.6, 5.0)
Routine & manual	15.4 (13.5, 17.3)	4.5 (2.4, 6.6)
Other*	14.8 (11.0, 18.5)	3.0 (-0.6, 6.6)
**Age 50–64 years by socioeconomic status**		
Managerial & professional	7.7 (5.4, 10.0)	2.5 (0.8, 4.2)
Intermediate	10.5 (7.7, 13.3)	2.7 (0.9, 4.6)
Routine & manual	13.6 (11.7, 15.4)	4.3 (2.6, 6.1)
Other*	17.9 (12.7, 23.1)	3.6 (-1.1, 8.2)
**Age 65–74 years by socioeconomic status**		
Managerial & professional	7.7 (4.6, 10.8)	2.3 (-0.2, 4.9)
Intermediate	10.0 (6.2, 13.8)	2.7 (0.1, 5.4)
Routine & manual	12.1 (9.9, 14.4)	3.8 (1.8, 5.7)
Other*	14.1 (8.6, 19.6)	2.7 (-1.7, 7.2)
**Age ≥75 years by socioeconomic status**		
Managerial & professional	7.0 (3.1, 10.8)	2.0 (-0.5, 4.5)
Intermediate	13.6 (8.5–18.8)	4.0 (0.3, 7.8)
Routine & manual	12.8 (9.8, 15.7)	3.9 (1.3, 6.4)
Other*	7.1 (3.2, 11.1)	0.8 (-0.7, 2.3)

* The ‘other’ category includes housewives and individuals whose occupational class could not be determined or was inadequately described. CI: Confidence interval; SR: Symptomatic radiographic; OA: Osteoarthritis.

### Sensitivity analysis of different thresholds of radiographic severity

Using a lower threshold of radiographic severity of grade≥1 for either osteophytes or joint space narrowing on either view the prevalence of symptomatic radiographic ankle OA to be 8.8% (7.9, 9.8). Whereas using a higher threshold of grade = 3 for either osteophytes or joint space narrowing on either view resulted in a prevalence estimate of 1.9% (1.0, 2.7). Irrespective of the threshold used to define radiographic OA, symptomatic radiographic ankle OA was still more prevalent in females, in younger individuals aged 50–64 years and in individuals identified as having routine or manual occupations (Table 4).

**Table 4:** Population prevalence estimates of symptomatic radiographic ankle OA using different grade of radiographic severity, stratified by socioeconomic status and age and gender.

	Proportion estimate for SR ankle OA (grade≥1): % (95% CI)	Proportion estimate for SR ankle OA (grade = 3): % (95% CI)
**Overall**	8.8 (7.9, 9.8)	1.9 (1.0, 2.7)
**Gender:**		
Males	6.5 (5.4, 7.7)	1.5 (0.7, 2.3)
Females	11.1 (9.7, 12.5)	2.2 (0.9, 3.5)
**Age (years):**		
50–64	9.0 (7.7, 10.3)	2.0 (0.9, 3.0)
65–74	8.5 (6.9, 10.0)	1.7 (0.6, 2.9)
≥75	9.1 (7.2, 11.0)	1.7 (0.6, 2.8)
**Males by Age (years):**		
50–64	7.2 (5.4, 9.0)	1.9 (0.7, 3.0)
65–74	5.6 (3.8, 7.4)	1.1 (-0.1, 2.3)
≥75	6.0 (3.5, 8.5)	1.1 (-0.3, 2.5)
**Females by Age (years):**		
50–64	10.8 (8.8, 12.8)	2.1 (0.5, 3.7)
65–74	11.4 (8.8, 14.0)	2.4 (0.8, 4.1)
≥75	11.4 (8.7, 14.1)	2.2 (0.2, 4.1)
**Socioeconomic status**		
Managerial & professional	6.2 (4.6, 7.9)	1.1 (-0.0, 2.2)
Intermediate	8.7 (6.6, 10.7)	1.4 (0.2, 2.7)
Routine & manual	9.7 (8.3, 11.0)	2.1 (0.8, 3.4)
Other*	9.6 (6.2, 13.0)	2.7 (-0.3, 5.8)
**Males by socioeconomic status**		
Managerial & professional	3.9 (2.2, 5.6)	0.6 (-0.4, 1.6)
Intermediate	7.0 (4.3, 9.7)	1.3 (-0.2, 2.7)
Routine/ manual	7.6 (5.8, 9.3)	1.9 (0.6, 3.2)
Other*	6.3 (1.9, 10.7)	1.9 (-1.1, 5.0)
**Females by socioeconomic status**		
Managerial & professional	9.5 (6.5, 12.6)	1.8 (-0.1, 3.7)
Intermediate	10.2 (7.2, 13.2)	1.6 (-0.2, 3.4)
Routine & manual	11.7 (9.8, 13.7)	2.3 (0.5, 4.0)
Other*	11.3 (7.0, 15.5)	3.1 (-0.6, 6.9)
**Age 50–64 years by socioeconomic status**		
Managerial & professional	6.1 (3.9, 8.3)	0.9 (-0.3, 2.2)
Intermediate	8.2 (5.4, 11.0)	1.6 (-0.0, 3.2)
Routine & manual	9.9 (8.0, 11.7)	2.2 (0.7, 3.6)
Other*	11.6 (5.8, 17.5)	3.9 (-1.1, 8.9)
**Age 65–74 years by socioeconomic status**		
Managerial & professional	6.6 (3.5, 9.7)	1.5 (-0.4, 3.5)
Intermediate	7.7 (4.0, 11.3)	0.9 (-0.7, 2.6)
Routine & manual	8.9 (6.7, 11.1)	1.9 (0.3, 3.5)
Other*	11.1 (5.5, 16.6)	2.6 (-2.1, 7.4)
**Age ≥75 years by socioeconomic status**		
Managerial & professional	6.1 (2.3, 9.9)	1.0 (-0.9, 2.8)
Intermediate	11.9 (6.8, 17.0)	2.0 (-0.7, 4.6)
Routine & manual	10.5 (7.7, 13.4)	2.2 (0.4, 4.0)
Other*	4.8 (1.4, 8.1)	1.0 (-0.8, 2.8)

* The ‘other’ category includes housewives and individuals whose occupational class could not be determined or was inadequately described. CI: Confidence interval; SR: Symptomatic radiographic; OA: Osteoarthritis.

## Discussion

Through both a systematic review and cross-sectional epidemiological study, this paper aimed to identify the population prevalence of radiographic ankle OA. The systematic review found that radiographic ankle OA has been previously under-researched in a community-dwelling setting. There was heterogeneity in the methods of assessing and defining the presence of radiographic ankle OA and general population prevalence estimates of radiographic ankle OA were not found within the existing literature. In our population of community-dwelling older adults aged 50 years and over, the period prevalence of 11.7% was found for ankle pain and 3.4% for symptomatic radiographic ankle OA. Stratified estimates found that the prevalence of both ankle pain and symptomatic radiographic ankle OA was higher in females, but lower in older age groups. Ankle pain and symptomatic radiographic ankle OA were also more prevalent in routine and manual occupations compared with managerial professions.

The systematic review demonstrates that there is little published literature available for ankle OA in the community-dwelling populations. In comparison, previous systematic reviews of studies examining radiographic OA in the general population have all found greater numbers of published studies pertaining to the hand (176) [8], hip (23) [6], and foot (27) [9]. In addition, there were 190 epidemiological studies that used the Kellgren and Lawrence grading system alone to assess knee OA [7]. This systematic review also showed that no consistent method of assessment for radiographic ankle OA has been used and consequently the definitions of ankle OA varied greatly. Reviews of radiographic OA at other joint sites have found the Kellgren and Lawrence scale to be the most commonly used method of assessment [6,8,9]. However, in the current systematic review only two studies used this scale [27,30], with the commonest form of assessment being the use of selected individual radiographic features. In addition to the limitations that have been noted for the Kellgren and Lawrence system [43] and variations in the interpretation of the scale [7], this could also be due to the recognition that the presentation of OA varies at different joint sites. For example, malalignment and erosion of the central cortical bone can be present in the hand joints [44], thickening of the medial femoral calcaneum and flattening of the femoral head at the hip, and medial tibial attrition at the tiobiofemoral joint [45]. To address these issues, joint-specific scoring scales and atlases have been developed and are available for the hands, hips and knee joints, and more recently, the foot. However, at the time of the review none were available for the ankle, except the Bargon Arthrosis score which is a global scoring system developed to assess post-surgical complications [33,40,44–46]. The other atlases identified in the systematic review had all been adapted for use in the ankle or adapted for use in OA [20,31,32].

The prevalence of radiographic ankle OA was infrequently reported by studies and none were applicable to a general population. Published prevalence estimates ranged from 0.0% to 97.1%, the range and very high estimates are reflected by the selected populations and comparator groups examined as well as differences in the ages groups examined and methods used to assess radiographic ankle OA. As most of the selected populations were associated with sporting groups, it is likely that the high prevalence estimates in these studies would be much higher due to increased injury rates leading to post-traumatic forms OA compared to general populations. Therefore, owing to the heterogeneity of populations, methods of assessment and definitions of radiographic ankle OA, an overall prevalence estimate could not be derived from the studies in this systematic review. Additionally, it was not possible to examine stratified prevalence estimates for factors such as age and gender, unlike previous reviews of radiographic foot [9], hip [6,47] and knee OA [48].

The systematic review highlights the novelty of the prevalence estimate of 3.4% for symptomatic radiographic ankle OA in a community-dwelling population provided by our study. This estimate is similar to the reported prevalences of self-reported ankle pain (assumed to be symptomatic ankle OA) in retired football players and self-reported ankle OA in a general Australian population of 2.6% and 4%, respectively. However, these are both point prevalence estimates which either lack a true general population sample or the use of statistical methods to extrapolate prevalence estimates back to the general population, as employed in our study [13,49].

When compared with symptomatic radiographic OA at the hand (15.0%), hip (6.2%), knee (12.0%) and foot (16.7%) [35,50,51], our prevalence estimate for symptomatic radiographic ankle OA (3.4%) is significantly lower. One reason for this may be differences in the anatomical and biomechanical properties of cartilage at the ankle joint, which may be more resilient compared to other joints [52]. Radiographic grade≥2, which was a required part of our definition of symptomatic radiographic ankle OA, is comparable to the definition used for foot OA [35] but is a higher threshold than tends to be used for radiographic knee, hip and hand OA and could explain our estimate at the ankle in comparison to these sites [50,51]. An estimate of 8.8% was obtained using a lower threshold of severity (grade≥1) for symptomatic radiographic ankle OA, which is stilll lower than estimates of hand or knee OA, but higher than hip OA. This suggests that our prevalence estimate of 3.4% using the cut-off of grade≥2 could be considered to be an estimate of moderate to severe symptomatic radiographic ankle OA. Previous estimates for ankle pain in general adult populations range from 9% [4] to 15% [5], although the populations in these studies were more restricted in age (25 years and over, and 65 years and over, respectively) and these studies employed different time periods over which the occurrence of ankle pain was captured (12 month and one month respectively).

The prevalence of both ankle pain and symptomatic, radiographic ankle OA was slightly higher in females than males. Increased prevalence of ankle pain in females as also noted in the previous ankle pain studies, but may reflect previous findings that females are more likely and willing than men to report pain [4,5,53–56]. This gender pattern, while not previously examined in the general population at the ankle, is observed in knee and hand OA but is less marked at the hip [50,57,58]. Gender differences could be due to referred pain or biomechanical consequences of other conditions such as hallux valgus and ankle sprains, both of which are also known to have a higher prevalence in females [59,60]. In addition, there is also the increased risk of OA identified in older women that is thought to be due to the reduction in post-menopausal oestrogen production [58,61]. The higher prevalence estimates of ankle pain and slightly higher estimates of symptomatic radiographic ankle OA in adults aged 50–64 years contradicts other studies which report a positive association between age and the prevalence of symptomatic radiographic OA [47,62,63,64]. However, decreases in prevalence of joint pain and OA in older ages has been reported, though less commonly [4,47,65]. Older age is unlikely to affect structural changes observed radiographically, but may affect the reporting of pain due to stoicism, altered perception of pain or poor recall [66]. However, it is also possible that there is more pronounced healthy cohort effect in older participants who took part in the study. No previous studies have investigated the prevalence trends for ankle pain and socioeconomic status, however the increased prevalence in routine and manual occupational classes found in this study is consistent with patterns seen for other musculoskeletal pain and OA [55,67]. This is likely to be due to the high physical demands and heavy workloads experienced by employees in manual and routine occupations.

While we examined the effect of person-level risk factors (age, gender and socioeconomic status) have on ankle OA prevalence there are other notable joint-level risk factors that should be acknowledged for ankle OA. Previous injury or trauma to the ankle or surrounding structures has been recognised as an important localised risk factor for the subsequent development of ankle OA [68]. However, this study was undertaken in patients from a tertiary orthopaedic department and may have been affected by selection bias. In addition to this, OA and malalignment at the knee have been associated with degenerative changes at the contralateral ankle [69]. This is thought to be due to the changes in alignment and weight distribution that occur secondary to the OA process as well as accompanying pain symptoms [69].

The overall prevalence of ankle pain (11.7%) is significantly higher than the prevalence of symptomatic, radiographic ankle OA (3.4%). Comparing prevalence estimates for definitions of joint pain and symptomatic radiographic OA at the knee also shows similar trends [70]. Similar results for the ankle joint are also demonstrated in a study investigating the prevalence of ankle pain and self-reported OA in a population of retired footballers [13]. It is possible that ankle pain may be attributable to other pathologies occurring at the joint site or in surrounding structures, for example ligamentous or tendon injury, ankle sprain, or referred pain from other areas. However, it is also possible that pathophysiological changes are present but that the OA bony changes visible on radiographs are not yet present or detectable as this imaging method is known to be less sensitive than other imaging methods [71]. Other more sensitive imaging modalities such as Magnetic Resonance Imaging (MRI) that allow the visualisation of soft tissue as well as more detailed views of the joint may identify higher prevalence estimates of ankle OA.

The strengths of this systematic review include a robust search strategy and application of inclusion and exclusion criteria. In addition, translation was used for non-English language papers rather than exclusion and authors of the publications were contacted where necessary to seek clarification. However, this systematic review used a single reviewer for title, abstract and full-text screening as well as quality appraisal and data extraction, increasing the risk of errors and bias. Additionally, publication bias was not considered as estimates of the prevalence of any condition are much less likely to be affected by publication bias as statistical significance is not a major consideration and we were not able to undertake any meta-analysis due to heterogeneity of the studies.

The cross-sectional secondary analysis included census sampling to recruit adults aged 50 years and over. This, along with the large sample size and similar age and gender distributions to the National Census 2011, gives greater confidence in the generalizability of the results of this study [35,71]. The standardised methods that were used to obtain weight-bearing AP and lateral views of the ankle and the scoring system including atlas were fully specified. A combined symptomatic and radiographic case definition was used and explicitly stated. Statistical methods used to account for missing data and non-response bias provided a full dataset for secondary analysis and allowed overall prevalence estimates to be calculated for the total eligible target population.

There are also a number of limitations which should be acknowledged. Most importantly, radiographic ankle data were only collected for those who reported pain in and around the foot in the last year. It is possible that not all responders may have perceived the ankle as being part of the foot. However, sensitivity analysis found that less than 2% of individuals who reported ankle pain on a whole body pain manikin in the last one month did not report pain in or around the foot in the last year (data not shown). Secondly, previous injury of the ankle was not examined in this study, and so the prevalence of ankle OA attributable to this a potentially major risk factor for ankle OA could not be determined [68]. Thirdly, the restricted number of variables used within the weighted logistic regression model may mean that other factors that could have influenced response to the initial baseline health survey questionnaire have not been accounted for. Fourthly, while the intra-rater agreement for the presence of ankle OA was excellent, inter-rater agreement was found to be fair. The markedly lower inter-rater agreement is comparable or only slightly lower than agreement that has been previously reported in a recently developed ankle atlas [72], the original foot atlas [40] and the assessment of foot OA in the same population. The inter-rater agreement does vary at large joint sites such as the hip and knee [73,74], but also in other small joint sites such as the hands [75]. However, the ankle may be particularly challenging to score due to difficulties eliminating talar tilt and rotation and it has been noted that the levels of severity can vary across different regions of the joint [72]. As the second assessor scored radiographs only for reliability purposes and only scores from a single reader, who had excellent intra-rater agreement, were used for the main analysis, differences in interpretation of the atlas would not have led to differential classification of ankle OA within the study. However, given that the single main assessor was more conservative in their approach to scoring, the population prevalence estimates may have been underestimated. Additionally, although the CASF study population has been shown to be representative of the UK general population, there is under-representation of ethnic minority groups when compared to proportions in the overall UK population [71].

In summary, from the findings of the systematic review it can be concluded that there is a lack of existing research on ankle OA within the community-dwelling setting and great heterogeneity was seen between these existing studies in the radiographic assessment and definitions for ankle OA that were used. Ankle pain affected one in nine individuals in a community-based sample of older adults whereas symptomatic radiographic ankle OA occurred much less frequently affecting approximately one in 29 individuals. Radiographic joint changes therefore only explain a small proportion of those with pain, further investigations of the prevalence of ankle OA using more sensitive imaging modalities are warranted.

## Supporting information

S1 File PRISMA 2009 checklist. (DOCX)

S2 File Radiographic classification atlas of ankle osteoarthritis. (PDF)
