# Supplementary material for: Population prevalence and distribution of ankle pain and symptomatic radiographic ankle osteoarthritis in community dwelling older adults: A systematic review and cross-sectional study
Source: PLoS One. 2018 Apr 30;13(4):e0193662. doi: 10.1371/journal.pone.0193662 (PMC5927448; doi:10.1371/journal.pone.0193662)
Supplement: S2 File — (PDF) [file pone.0193662.s002.pdf]

Radiographic Classification Atlas of Ankle Osteoarthritis

- Scoring system
- Ankle (anterior-posterior): osteophytes
- Ankle (anterior-posterior): joint space narrowing
- Ankle (lateral): osteophytes
- Ankle (lateral): joint space narrowing

Scoring system

Osteophytes

- 0 - absent
- 1 - small
- 2 - moderate
- 3 - severe

Joint space narrowing

- 0 - none
- 1 - definite
- 2 - severe
- 3 - joint fusion at least one point

Case definition of foot osteoarthritis in individual joints

- Radiographic OA can be considered to be present if a score of 2 or above is documented for either osteophytes or joint space narrowing, from either the anterior-posterior or lateral view

Anterior-posterior view - osteophytes

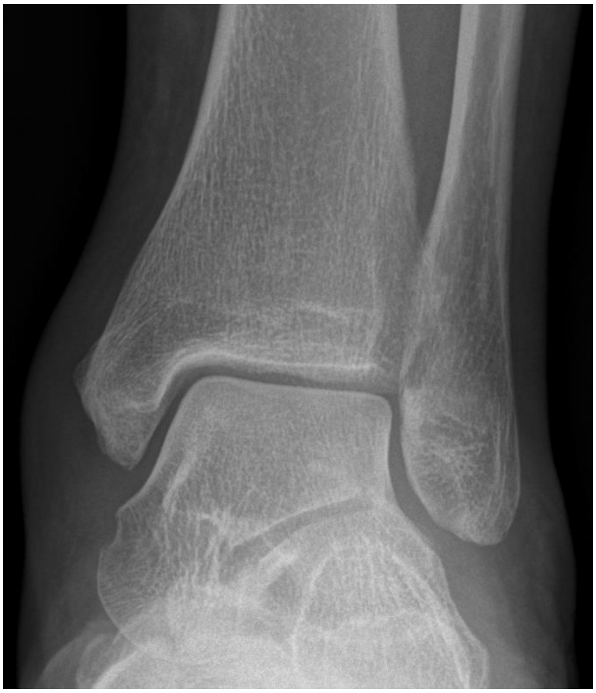

0

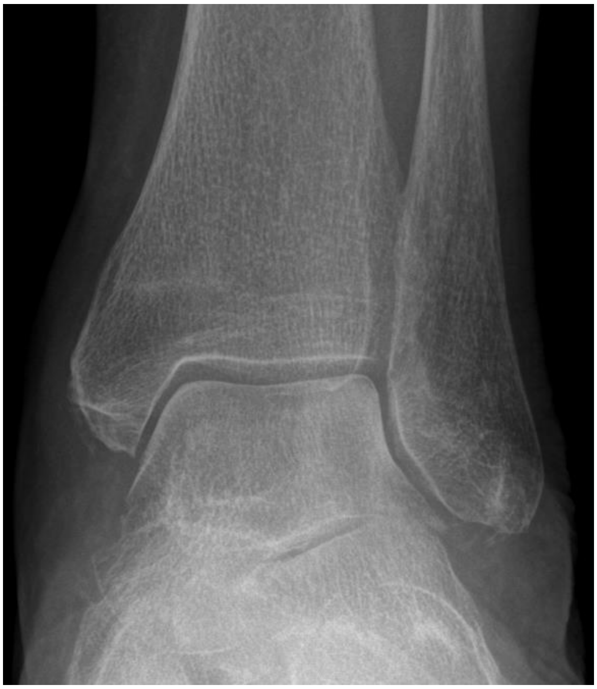

1

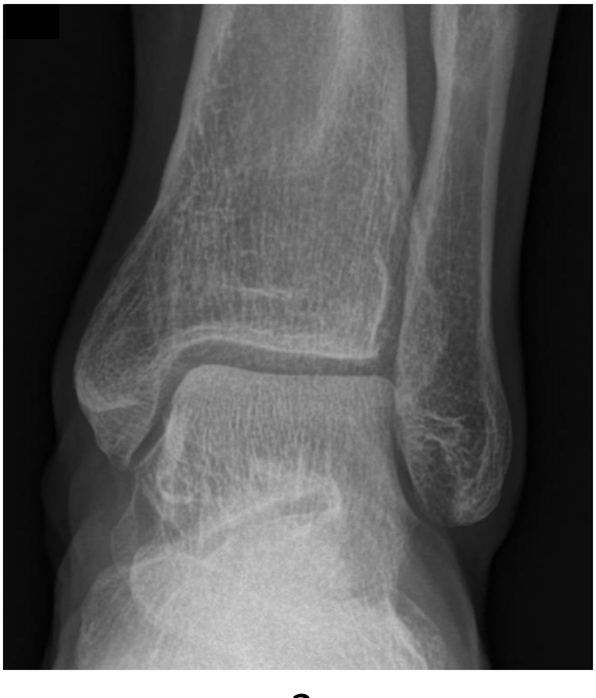

2

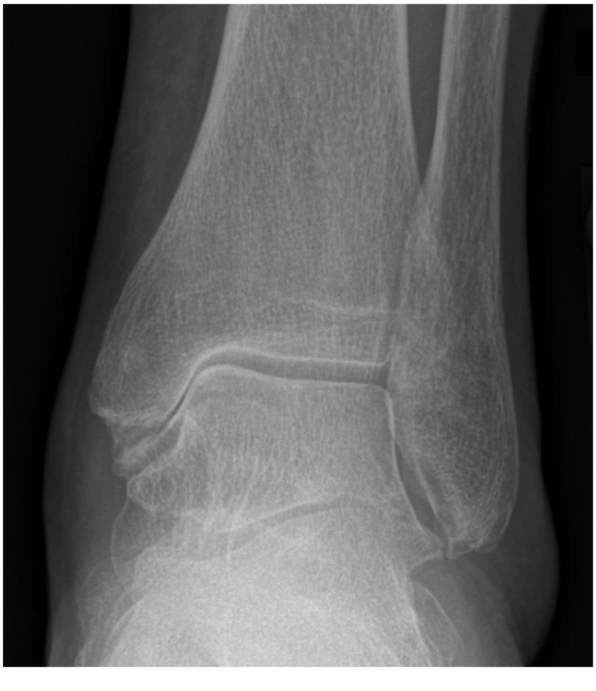

3

Anterior-posterior view – joint space narrowing

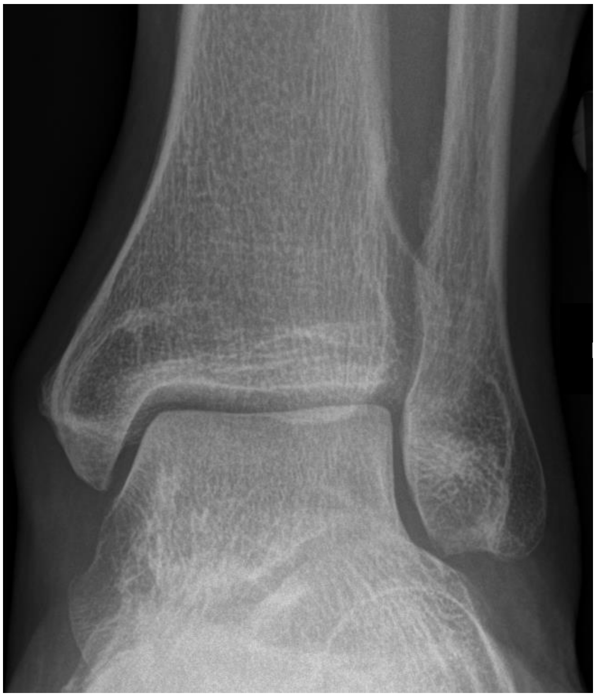

0

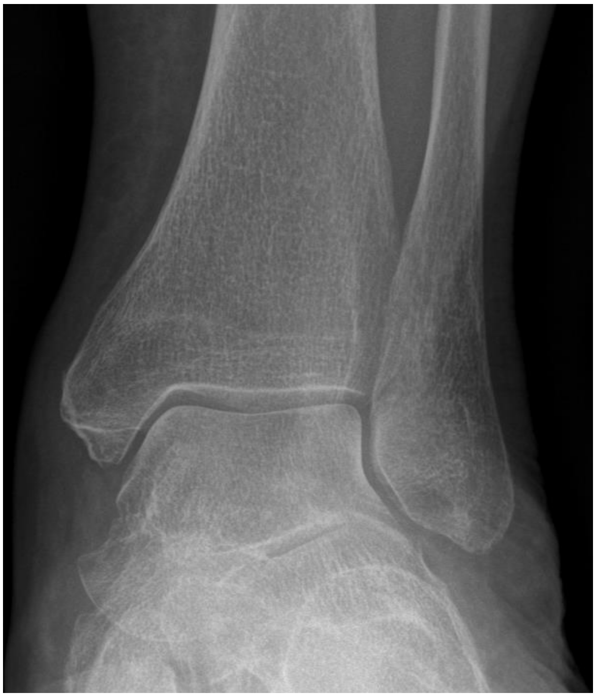

1

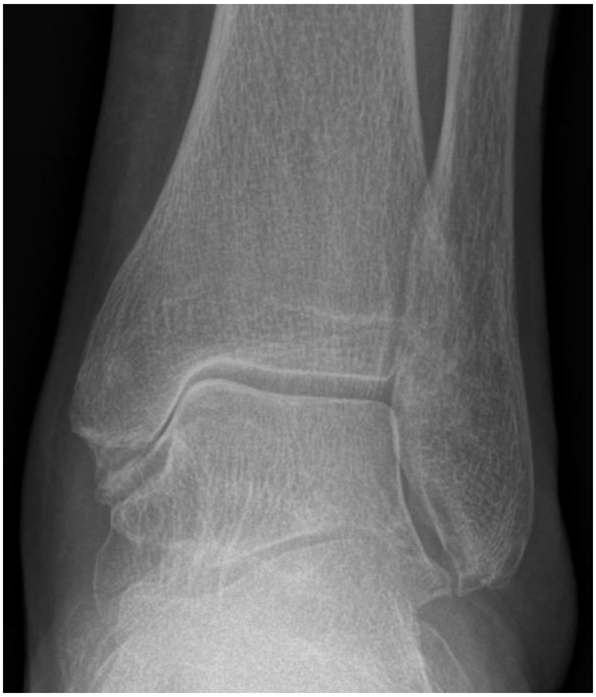

2

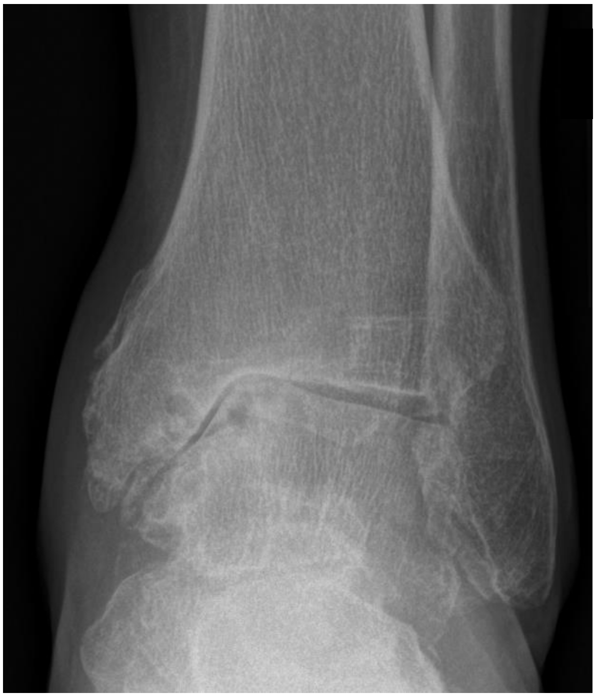

3

Lateral view - osteophytes

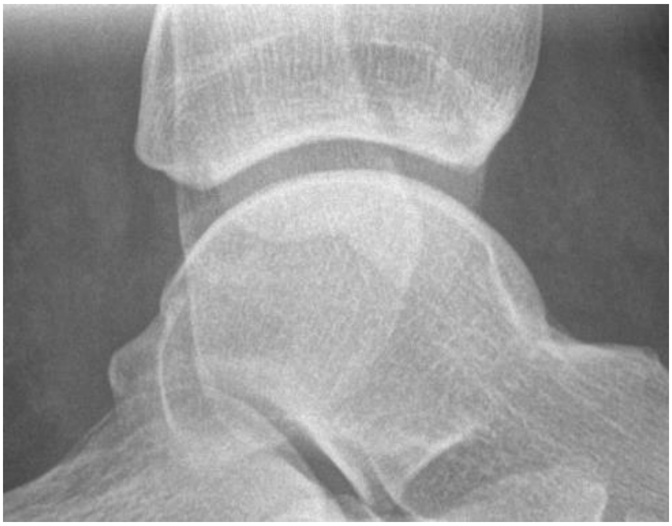

0

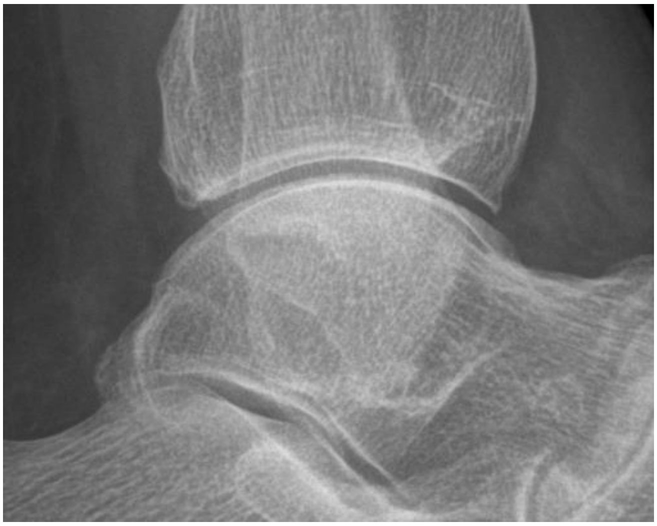

1

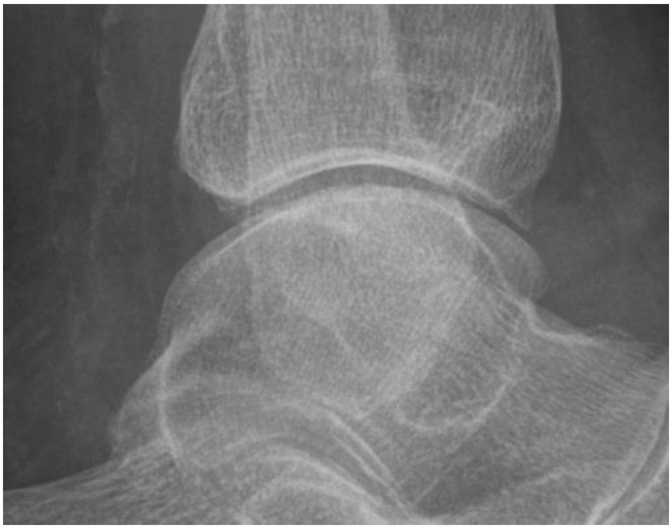

2

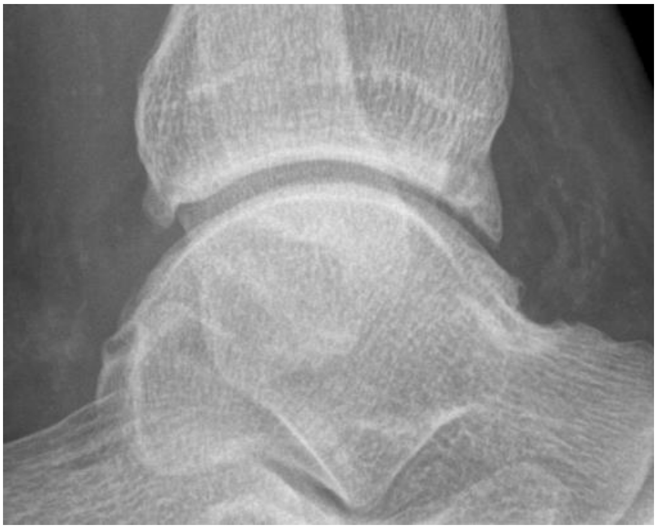

3

Lateral view – joint space narrowing

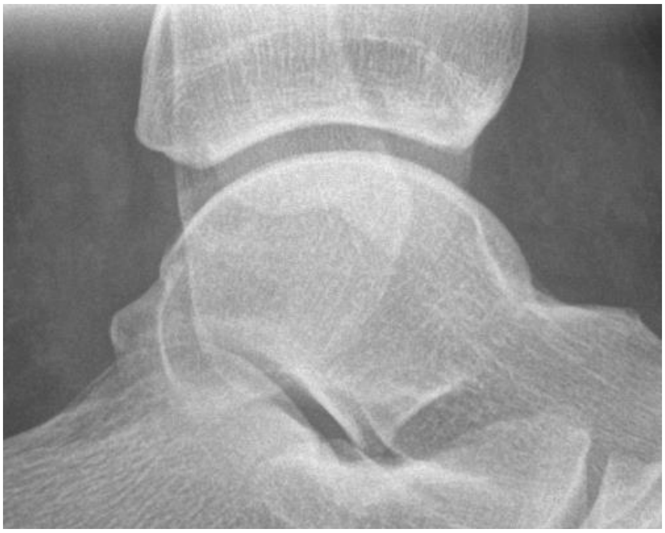

0

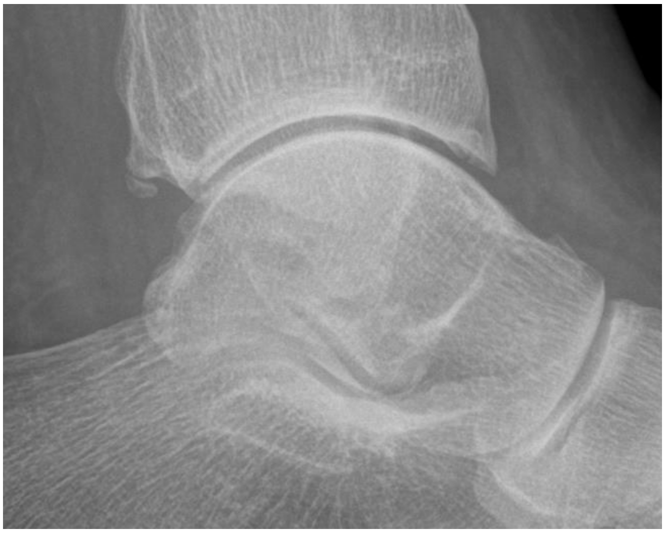

1

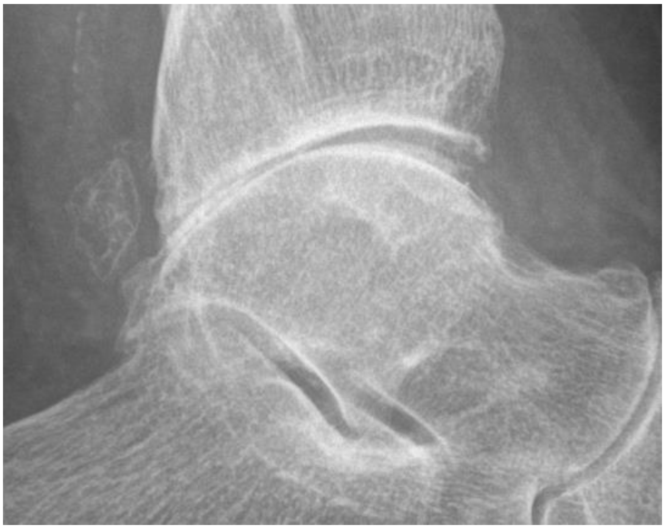

2

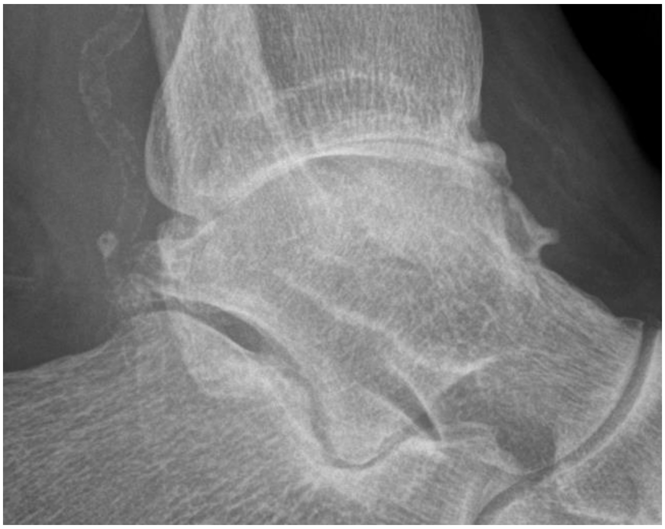

3
